# The Access to Antenatal and Postpartum Care Services of Migrant Workers in the Greater Mekong Subregion: The Role of Acculturative Stress and Social Support

**DOI:** 10.1155/2018/9241923

**Published:** 2018-03-01

**Authors:** Charamporn Holumyong, Kathleen Ford, Siriporn Sajjanand, Aphichat Chamratrithirong

**Affiliations:** ^1^Institute for Population and Social Research, Mahidol University, Salaya, Thailand; ^2^School of Public Health, University of Michigan, Ann Arbor, MI, USA; ^3^Faculty of Business Administration, Bangkokthonburi University, Bangkok, Thailand

## Abstract

The objective of this paper is to determine whether social support and acculturative stress were related to obtaining antenatal and postpartum care for pregnant female migrants, as well as access to health care for migrant children. The study utilized data of 987 migrant workers in Thailand who originated from hill tribes and mountain communities in Myanmar and Cambodia. Regression analysis showed that the language barrier, a crucial factor behind acculturative stress, adversely influenced access to maternal care. Social support reduced the impact of acculturative stress. Migrants with support are more likely to access health care. Based on the Multidimensional Scale of Perceived Social Support, more sources of support either from friends, family members, or other supporters who are significant could increase health care access. Besides friends and family, the support from the Migrant Health Worker Program and Migrant Health Volunteer Program allowed the formal health sector to utilize the informal social networks to improve care for migrants.

## 1. Introduction

The Greater Mekong Subregion (GMS) comprises six countries of the Mekong River basin: Cambodia, Lao PDR, Myanmar, Thailand, Vietnam, and China (i.e., Yunnan Province). Despite being connected by the Mekong River, parts of the GMS are characterized by elevated terrain, as it encroaches upon the Himalayas. The mountain population of this subregion lives at the margins of society. The rugged, hard-to-reach mountainous areas include populations living in poverty and poor health conditions [1]. The mountain states of Myanmar, one of the poorest nations of the region, had a higher level of poverty than other states, including food poverty [2]. In Cambodia, more than 40% of poverty areas were in provinces in the Plateau/mountain zone [3].

Migration in the GMS has been an important phenomenon for improving migrant quality of life, and Thailand has been a popular destination for low-wage labor in the region for more than three decades [4]. Individuals who inhabit the mountains (as well as their accompanying dependents) have been among those groups of people seeking a new life with better economic opportunities in Thailand. In 2014, about half (53.9%) of Burmese migrant workers in Thailand migrated from the mountainous region of Myanmar (e.g., northwest of Chin state, northwest of Rakhine state, northeast of Kachin state, and northeast of Shan state). For Cambodian migrants, more than two-thirds (72.2%) migrated from the Plateau/mountain zone in 2014. Working and living conditions of the mountain populations differed slightly from other groups of migrants in Thailand. Most of their work in Thailand is unskilled and poorly paid.

It is estimated that, in 2015, there were 4.19 million migrants from Myanmar and Cambodia working in Thailand [5]. Many are temporary migrants working in the construction, agriculture, fishing, and industrial sectors. These migrants are motivated by a lack of economic opportunity in their home country and the relatively higher wages and better working conditions in Thailand. Much of this migration can be characterized as “irregular migration” as many of these migrants do not have proper legal documents or work permits [6]. They rely on extensive networks of relatives and friends to facilitate border crossing and obtaining jobs in Thailand [7]. These workers are an important component of the low-skilled labor force and are helping promote the expansion of the Thai economy. However, this type of migration makes them vulnerable to trafficking, fraud, or exploitation. Furthermore, undocumented migrants in Thailand cannot access social protection and basic rights [8], and this is a source of stress and anxiety for the migrants and a burden for Thai government services. The health care system for migrants is a contentious public service issue. There is a fear that the high number of migrants that are being served at health centers displaces local Thais. There is also concern that service provision to migrants could increase expenses of the attending hospitals since many migrants do not have health insurance. In fact, documented migrants are eligible for Thai health insurance but must buy it annually [9]. Also, it is assumed that undocumented migrants are not covered by any public health insurance scheme. In practice, they are responsible for paying for medical expenses in case of illness. Thus, self-medication and out-of-pocket payment for private care is a common practice for many migrants [10].

There is some evidence that immigration may benefit health. A person may make better use of health care, when migrating to areas with good health facilities [11]. Therefore, moving out from the mountain communities to the low-land urbanized areas in Thailand should improve accessibility to public health services. That access would be especially important for pregnant women and young children. On the other hand, access to health services is also influenced by culture, economic status, social networks, and community integration, which may pose barriers to care for newcomers [12]. This study, therefore, aims to analyze the role of different factors affecting accessibility to Thai maternal and child health (MCH) care for migrants from Myanmar and Cambodia who originated from hill tribe and mountain communities.

Previous research on migration and health has identified several factors related to MCH access. Financial costs have often been noted as an important barrier to accessing health care. In Ghana, the cost of care negatively influenced antenatal clinic attendance of pregnant women [13]. In Canada, immigrant women were more likely to be in the lower socioeconomic group and were more at risk of postpartum depression, but they were less likely to receive financial aid [14].

Together with the socioeconomic status of migrants, language and cultural barriers also influence the lack of MCH access of marginalized population. Cultural barriers from both providers and users of care have essentially influenced migrants' health access. On the supply side, the differences in health care services are rooted in the historical racial exclusion, discrimination, and contemporary social inequality [15]. Discrimination in health care could be embodied in organizational leadership, structural process of care, and attitudes of health staff including physicians, health experts, and nurses [16, 17]. Disdain toward lower-income migrants results in lack of institutional support policy and unwelcoming care, longer service waiting times, and subconscious prejudice of providers.

In the context of users of the services, health beliefs, values, preferences, and behaviors often steer migrants to substandard care providers. Dietary and hygiene practices, drug, alcohol, and tobacco use, and stress management are rooted in cultural norms [18]. Health status, therefore, can vary by race and ethnicity. For example, the prevalence of blindness and visual impairment among blacks is higher than for whites [19]. Asians tend to have poorer health than Caucasians based on their perceived health status reports [20]. Previous research has shown that diverse health beliefs impact health outcomes. Ford and Chamrathrithirong [21] found that perceived AIDS risk of migrants in Thailand influenced the use of condoms. Greenhalgh et al. [22] pointed to the beneficial effects of a deterministic attitude to prognosis of diabetes in Bangladeshi patients on their dietary adjustment and treatment cooperation. Based on their health-seeking behavior, previous studies have pointed to the low rate of care usage and access to care at the later stages of illness among minorities [23, 24]. Lanoy et al. [25] showed that migrants tended to delay seeking care for HIV infection in France compared to nonmigrants. Peng et al. [26] found that migrants' health care access was influenced by an unsupportive health service system in China. Migrants were discouraged from seeking appropriate care without an affordable health care service system.

The cultural and social patterns of hill people vary across tribes. Each tribe has its own language and tradition [27]. Shamanism and beliefs in traditional spirits have been part of their culture for countless generations. Rituals play a significant role in seeking good health [28] and spirit offerings are routinely practiced by women who pray for good health of family members [29]. Despite the limited studies on health status of hill people in the GMS, the findings of the existing research point to serious lack of coverage of MCH among this group, resulting in malnutrition of women of reproductive age, miscarriage, neonatal death, and underweight mothers and infants [30, 31].

Acculturation is broadly defined as the process of cultural and psychological adjustment that leads to a change of one's cultural identity driven by the assimilation of at least two groups of cultures [32, 33]. When arriving in the destination community, migrants are immersed in a new environment which entails interaction with the host community. Acculturation is, therefore, important to a migrant's survival, and interacting with people of different cultures leads migrants to assimilate to the cultural norms of the host society. Under the process of acculturation, migrants often encounter the feeling of distress from a sense of loss, dislocation, marginalization, alienation, and isolation [34]. Stress from acculturation was also found to have a significant negative impact on health care access. Frisbie et al. [35] found that Asian and Pacific Islander adults in the US seemed to have less access to formal medical care than Americans. Yi [36] studied access to health care of Vietnamese women in the US and found that length of residence and language ability affected access to care. An increase in length of residence and English language proficiency led to better access to health care among migrants in the US. Sentell et al. [37] found racial/ethnic disparities in mental health care. Limited English proficiency, especially among Asian, Pacific Islanders, and Latinos, was associated with lower use of mental health care. Besides language, Jang et al. [38] studied the influence of citizenship on the use of health care services by Chinese-Americans.

Acculturation is equated with ability to adjust, adapt, and integrate with the host community. The acculturation process can vary by sociocultural, economic, and geographical circumstances. Berry [33, 39] has segregated typologies of integration or acculturation based on the capacity to adjust and maintain one's own culture which includes full integration, separation, assimilation, and marginalization. Successful integration of migrants has to include active social interrelationships with natives while preserving one's own cultural identity and practices. With the studied population, it is observed that Burmese and Cambodian migrants are not fully integrated into Thai society. The majority of them remain confined to a marginalization situation [6]. Only a minority of Burmese and Cambodian migrant workers participate in local activities with Thais such as important Thai religious days, important religious days of the home country, merit-making, Thai cultural events, or community social events.

Social support has been known to mitigate acculturative stress [40]. Family, friends, and other persons who play a significant role constitute important sources of material aid as well as motivational support in accessing health services. We posit that previous experiences of migrants in receiving social support on health care from family and friends will result in improved access to MCH services.

Employers and health staff, including migrant health workers (MHW) and migrant health volunteers (MHV), are considered to be an important bridge between migrants and the formal health care system. In addition, many migrants live in the workplace and have close relationships with their employers beyond supervisor-employee relationships. In the Thai culture, employers provide other types of support to their migrant workers such as advising on life issues, saving money for their workers, and monitoring their workers' health. Therefore, we considered employers as an important source of support who could influence migrants' access to MCH care.

MHW and MHV were also included in the group of supporters who influence access to MCH care among migrants. The MHW and MHV could be considered as an innovative service system that works to help migrants access quality health care in Thailand. Both MHW and MHV are considered as agents who could effectively and appropriately improve access to care among migrants with language and cultural barriers. MHW and MHV also perform as medical assistants who monitor disease in migrant communities [41]. Their services include knowledge dissemination on health issues and infectious diseases. With the support of MHW and MHV, the quality and quantity of services to migrants could be significantly improved [42]. Health facilities located in areas with a large concentration of migrants may hire and train MHW using funds from the Compulsory Migrant Health Insurance Scheme [43]. Despite improving access to care of migrants, the system of MHW and MHV is only an alternative option for health facilities. Therefore, only some facilities utilize this system while most leave migrants to overcome the barriers to care on their own.

The objective of this paper is to evaluate the role of socioeconomic status, acculturative stress, social support from family friends, and assistance from health volunteers and health professionals in influencing the use of health care of migrants who have moved from remote mountainous areas to Thailand. We hypothesize that socioeconomic status will be positively related to health care access and that acculturative stress will reduce health care access. Furthermore, social support from friends and assistance from health volunteers and health professionals will help to reduce acculturative stress and promote use of health care. Given the different environmental contexts and background characteristics between Burmese and Cambodian migrants, we hypothesize that country of origin of migrants could influence access to MCH.

## 2. Methods

The data were drawn from a 2014 sample survey undertaken in 17 provinces for the evaluation of the Prevention of HIV/AIDS among Migrant Workers Thailand Project (PHAMIT-2) conducted by the Institute for Population and Social Research of Mahidol University, in collaboration with the Raks Thai Foundation. The survey was financially supported by the Global Fund to Fight AIDS, Tuberculosis and Malaria. Migrant workers from Myanmar, Cambodia, and Lao PDR who had resided in Thailand for at least three months were the target respondents. The geographic location of the sample was chosen with consideration for the size of the migrant population, and the nationalities and occupations of the migrants. An estimate of the size of the migrant population in each of 17 provinces was made, and locations were selected with probability proportional to size (PPS). A snowball sampling technique was used due to the undocumented status of many migrant workers. Index (seed) respondents from various locations in the sampled area were chosen. Index respondents referred the survey team to other potential respondents. Each index line was limited to ten individuals. A total of 3,555 persons were interviewed.

Structured questionnaires were used in the survey. The questionnaire was translated into four languages (Thai, English, Cambodian, and Burmese). Respondents were interviewed by trained interviewers, who could speak both Thai and the native language of the migrants. Internal review board approval was granted by the Mahidol University Ethical Review Committee.

The 987 migrants who lived with children and originated from the mountain areas of Myanmar and Cambodia were selected for this analysis. Lao migrants were excluded from the analysis due to the small number of persons interviewed.

The first dependent variable is* access to antenatal care *(ANC)* and postpartum care *(PPC). Data were coded to indicate if respondents (1) were in the reproductive age (15–49 years) and married (Yes/No), (2) had a child or children (Yes/No), and (3) whose youngest child was aged 0–4 years (Yes/No).

The second dependent variable is* access to child health care*. Data were coded to indicate if respondents (1) were in the reproductive age (15–49 years) and married (Yes/No) and (2) had a child or children (Yes/No). Access to child health care was assessed by whether the respondent had a plan for quick and easy access to a health center or government hospital to take their child in the event of sickness.

Independent variables include* social support*. Sources of support in this study were identified by the Multidimensional Scale Perceived of Social Support (MSPSS), which grouped the sources of support into (1) family, (2) friends, and (3) others who play a significant role [44]. To observe its influence on migrants' access to MCH care, migrant experience of receiving support from family members, friends, and others for access to health care was included in the analysis. Support items are coded to indicate if the respondent had previous experience of being assisted by these three sources in going to the governmental health facilities as well as other forms of support on health care, such as transfer services, condom use distribution, and knowledge on HIV/AIDS, and coded as “ever received support” or “never received support.”

- Spouse and relative support were considered as the source of social support in the family group.
- Colleagues, neighbors, and friends were considered as the source of social support in the group of friends.
- Employers and health staff, including MHW and MHV, were considered as sources of social support in the group of “others playing a significant role.”

Support items are combined in a single-scale score to increase their internal consistency. The support is coded as follows: (0) never received support, (1) ever received support from one source either family, friend, or others, (2) ever received support from two sources of support, and (3) ever received support from three sources of support.

The unique aspect of MSPSS on the inclusion of support from a significant other allows us to investigate the role of employer, MHW and MHV. Psychometric characteristics of MSPSS with the reliability, factorial validity, and subscale validity have been demonstrated across a number of different samples including pregnant women, South Asian migrants, and Arab immigrant women [45–47]. However, the shortcoming of the study is that no research has been published on the psychometric properties of the MSPSS when used with migrant workers in the GMS.

The second independent variable is* acculturative stress*. To study the influence of acculturative stress on the access of MCH care, we included four indicators of a migrant's cultural assimilation in Thai society: duration of residence in Thailand (months), capacity to speak Thai (none, weak, moderate, fluent), possession of a Thai work permit (yes or no), and country of origin (Myanmar or Cambodia). The first three were used as indicators of acculturation that influenced access to health care in previous research [35–38]. The last indicator, country of origin, was included in this analysis as it indicates the original cultural identity, and that could affect the process of assimilation.

However, Burmese and Cambodian migrants were varied in their pattern of migration, lifestyle, social integration, and adaptation. Social support may differently impact access to health care depending on being Burmese or Cambodian. Therefore, the interaction between support and country of origin in predicting access to ANC, PPC, and child health care was investigated. The results indicate no interaction in predicting access to ANC and child health care. For access to ANC, The Wald Chi-square value is 1.89 with *p* = 0.39. For access to child health care the Wald Chi-square value is 7.62 with *p* = 0.22. Nevertheless, the results indicate significant interaction between support and country of origin in predicting access to PPC, and the interactive variable was included in the PPC model.

A third independent variable category was* Demographic and Socioeconomic factors* including gender, age, marital status, child living arrangement, education, occupation, and estimated daily wage.

- Gender: data were coded as male or female.
- Age was treated as continuous variable: data were recorded based on respondents' age at the time of the interview.
- Marital status was segregated into three groups: (1) married, spouse present, (2) married, living separately, and (3) divorced or widowed. Migrants who were single were not included in the analysis.
- The child living arrangement was controlled in the child health access model. The variable was segregated into three groups: (1) child lives with both parents, (2) child lives with the mother, and (3) child lives with the father.
- Education was segregated into four groups: (1) no schooling, (2) some primary, (3) primary, and (4) secondary or higher.
- Occupation was segregated into seven groups: (1) deep-sea or coastal fisherman, (2) fisheries-related, (3) factory worker, (4) commerce, (5) construction, (6) day laborer or domestic worker, and (7) agricultural work.
- Estimated daily wage was segregated into three groups based on the minimum wage law in Thailand which has set the minimum at 300 baht per day (approximately USD 9.3): (1) less than the minimum wage, (2) the minimum wage, and (3) more than the minimum wage.

Multiple logistic regression analysis was used to analyze the factors related to receiving ANC, PPC, and health care for children of migrants. Four groups of factors were analyzed and controlled in all models: demographic characteristics, socioeconomic factors, acculturative stress, and social support.

## 3. Results

The respondents' demographic profile is shown in Table 1. The data include 987 migrants from the mountain community and highland areas of Myanmar (65%) and Cambodia (35%) who had children. Respondents consisted largely of women and a large proportion of middle-aged workers. Almost 90% of migrants were married and lived with their spouse. Perhaps not surprisingly, more than four-fifths of migrants had equal, or less than, a primary education (84%). The proportion of migrants with no schooling was higher among Cambodians compared to the Burmese. Many of migrants worked in the “3Ds” jobs (“dirty, dangerous, and demeaning”) in the fishing industry and the construction industry. Cambodian migrants were clustered in the fisheries-related sector and the Burmese were clustered in the manufacturing sector. Over half of Burmese migrants (68%) earned at least the daily minimum wage. However, the majority of Cambodians earned less than the minimum wage (74%).

Descriptive statistics on acculturative stress and social support on health care access are also shown in Table 1. The largest proportion of migrants in this sample (65%) migrated from Myanmar and they are the majority group of migrants in Thailand generally. The migrants were relatively new to the country and had a low proficiency in speaking Thai. Under half (44%) could not speak Thai at all and one-third (32%) could speak Thai only weakly. A majority of Burmese (80%) possessed Thai work permits but only 7% of Cambodian had work permits. When migrants got sick or had to visit health facilities, the largest source of support was their family (63%) with a smaller proportion citing health staff (13%), friends (12%), and employers (8%). Support from others who are significant like the MHW, MHV, and employers are relatively more important for Cambodians compared to their Burmese counterparts. On the other hand, support from family and friends was more important for Burmese than for Cambodians.

The use of ANC and PPC was quite high among migrants. Fully 80% reported getting ANC during their latest pregnancy and 64% received PPC for their last birth. Correspondingly, a large proportion of migrants (84%) reported being able to take their child to a government health facility if they become sick. The majority of migrants' children lived with both parents (90%) and about half were aged 0–5 years (47%).

Table 2 shows the adjusted odds ratios derived from multivariate logistic regression analysis for receiving ANC during the migrants' last pregnancy. This analysis controlled for demographic characteristics, socioeconomic factors, acculturative stress factors, and social support factors. Acculturative stress, social support, and socioeconomic factors were significantly related to receiving ANC. The demographic factors were not significant. Language capacity positively influences access to ANC. Compared with migrant parents who could not speak Thai at all, those who were fluent in Thai were almost eight times more likely to receive ANC (AOR: 7.93). Considering the role of social support, a larger number of sources of support increase access to ANC (AOR: 12.17). Migrants with some education are more likely to receive ANC compared to those with no schooling. Among the acculturative stress and social support factors, country of origin, duration of migration, and having a work permit were not significantly related to ANC access.

Table 3 shows the factors related to receiving PPC for migrant mothers after last delivery. Both acculturative stress and social support factors were significantly related to receiving PPC. In Model 1, country of origin and social support were related to access to PPC. Compared with migrants from Myanmar, Cambodian migrants were significantly more likely to receive PPC (AOR: 0.10). The capacity of parents to speak Thai was also significantly related to PPC access. Those who were fluent in Thai were three times more likely to receive PPC compared with migrant parents who could not speak Thai at all (AOR: 2.94). Social support increased access to PPC. For socioeconomic factors, occupation was related to receiving PPC. Construction workers and merchants were less likely to receive PPC compared to agricultural workers.

Model 2 shows the significant role of interactive variables between social support and country of origin on access to PPC. Social support is crucially important for Cambodian migrants. Compared with migrants with no support, Cambodian migrants with at least one source of support were significantly more likely to receive PPC (AOR: 10.58).

Considering factors related to child health access among GMS migrant workers shown in Table 4, acculturative stress of parents, social support, demographic, and socioeconomic factors of parents were significantly related to child health access. Compared with migrants from Cambodia, the Burmese (AOR: 3.04) were significantly more likely to receive access to child health care. Social support on health from friends, family members, and health staff remains significant in determining child health care access. The increase in number of sources of support could increase child health access. The completeness of family structure can positively support child health access. Families with both mother and father present were more likely to have access to child health care compared to single-father families. Among socioeconomic factors, compared with agricultural workers, deep-sea and coastal fishermen (AOR: 0.06), factory workers (AOR: 0.04), and those working in commerce (AOR: 0.08) were significantly less likely to have access to child health care.

## 4. Conclusions

This study exposed loopholes in MCH care access for migrants who originated from hill tribes and mountain communities. Rooted with the unique characteristics of their original cultures, values, and beliefs, migrants who originated from hill tribes and mountain communities have different health behavior and also different experiences in access to care compared to other population groups. Our findings confirm the importance of acculturative stress and social support in use of MCH care among this group of migrants.

Most migrants who originated from hill tribes and mountain communities were in the early reproductive years. Many had low levels of education, and their work was concentrated in unskilled and poorly paid jobs. Despite the language barrier, the use of ANC, PPC and access to child health service was quite high. A high level of health care access has been partly influenced by the governmental master plan to improve migrant health, which was initially launched in 2007. A variety of programs have been implemented to reduce barriers to migrant access to health care, such as the Border Health Development Plan, the National Master Plan for HIV/AIDS Prevention, and the Care and Support for Migrants and Mobile Populations program, among others [48]. However, a specific program to improve migrants' access to MCH services has not yet been implemented. Among migrant workers from the mountains, one-fifth of pregnant women did not receive ANC and about one-third did not receive or seek PPC. Though the majority of migrant parents reported knowing where to receive health care for their children, about one-sixth remain uninformed.

Acculturative stress had an important effect on access to MCH services. Thai language proficiency is an essential factor determining migrants' access to MCH services in Thailand. The results showed that capacity to speak Thai had an important effect on obtaining ANC and PPC. Strong Thai proficiency may have helped migrants to learn the proper channels to receive health care services. Communication that leads to the exchange of useful knowledge also involves nonverbal communication [49]. Hill people may experience a different process of acculturation during migration compared to other groups of migrants. The outer display of lifestyles by migrants who originated from hill tribes and mountain communities could be stigmatizing which could also lead to discrimination and social exclusion. Low language proficiency reflects these migrants' limited ability to communicate with others and less absorption of the mainstream culture and lifestyle in the host society. Migrants who could speak Thai fluently were more likely to receive useful information, more likely to integrate in the community, and more likely to have a social network that could help them access health care.

Compared to those from Myanmar, migrants from the mountains of Cambodia were less likely to access health care for their children. This may be due to the dominance of migrant workers from Myanmar in Thailand, which creates a more “Burmese-friendly system.” Many local hospitals in areas with a large concentration of migrants implement outreach programs to the Burmese community [43]. Many private and public hospitals have signs in Burmese alongside Thai signs. There are Burmese translation services in many public hospitals and health centers, which could help improve access to health services of the Burmese migrant women and children relative to the Cambodian women and children (who do not have those services).

Social support is a crucial factor that significantly improved migrants' access to MCH services. Simultaneously, such support relieves the impact of acculturative stress on reducing the use of health care among migrants. Cambodian migrants who had at least one source of support were more likely to receive PPC compared to migrant with no social support. This phenomenon could be attributed to the different background characteristics between Cambodian and Burmese migrants. The Cambodians are relatively new settlers compared to their Burmese counterparts. The majority of Cambodian migrants are undocumented and are concentrated in difficult-to-integrate types of occupations such as deep-sea or coastal fisherman, fisheries-related jobs, and self-employed commerce. These factors could be barriers to integrating Cambodian migrants into the health care system. Therefore, social support is crucially important for Cambodian migrants to receive essential health services including PPC. Support from others who are significant, for example, MHW, MHV, and employers, is relatively more important for Cambodians compared to the Burmese. A larger proportion of Cambodians reported receiving help from significant others. The findings indicate the role of cultural context on immigrants; immigrants come in many shapes and forms. The impact of acculturation, integration, and social support can vary by community and group of migrants based on their cultural context. The cultural context not only impacts on MCH access of migrants but also affects their health outcomes. However, this study examined only the disproportionate access to MCH services. The findings may not apply to the different health outcomes among migrants in Thailand, and that is a limitation of the study.

Support from health staff improved migrants' access to health care. Health staff were also key persons who positively supported migrant's access to health care. The Migrant Health Worker Program and Migrant Health Volunteer Program were effective in reducing language and cultural barriers to obtaining public services. Moreover, the MHV is part of the outreach program to increase migrant knowledge on health and improve health conditions [50]. The volunteer system was effective in using the informal social network to draw migrants into the formal public health service system.

The analysis of socioeconomic factors found disparities in access to care for migrants with different occupations. This finding points toward the need to focus on outreach programs that suitably fill gaps in health care for certain occupational groups. Deep-sea and coastal fishermen and factory workers were less exposed to local communities. Seafarers spend many days and nights on boats, while factory workers are confined by the high-wall fences in the private areas of their factories. These groups were only minimally attached to local communities and may have received less information on health care compared to other occupational groups. Employers played an indirect, but essential, role in improving the migrants' access to care. Unsupportive workplace policies could lower migrants' ability to obtain public services and reduce access to useful information. In many cases health personnel were not allowed to enter some factories and fishing boats to provide outreach services. Some employers prohibited their migrant workers to have contact with NGO staff or MHV. Such policies hindered migrants' access to public services. Workplace policies that lower the barriers to health care access, especially in the fishing and manufacturing industries, could promote migrant's health. Health programs which have active participation by employers could improve migrants' MCH access, such as collaboration of employers with NGO staff or health personnel to provide advisory services and information on reproductive health for migrants.

## Figures and Tables

**Table 1:** Sample characteristics.

Characteristics	Myanmar	Cambodia	Total
Number of sample	642	345	987

*Demographic factors of respondents*			
Female	66.2%	77.4%	70.1%
Age (years)			
15–19	1.2%	1.4%	1.3%
20–24	9.3%	13.0%	10.6%
25–49	88.6%	84.1%	87.0%
>49	0.8%	1.4%	1.0%
Marital status			
Married, spouse present	88.9%	91.0%	89.7%
Married, living separated	6.9%	6.7%	6.8%
Divorced or widowed	4.2%	2.3%	3.5%

*Socioeconomic factors of respondents*			
Education			
No schooling	5.9%	25.5%	12.8%
Some primary	33.5%	27.5%	31.4%
Primary	44.9%	29.6%	39.5%
More than primary	15.7%	17.4%	16.3%
Occupation			
Deep-sea or coastal fisherman	7.0%	17.4%	10.6%
Fisheries-related worker	19.9%	54.2%	31.9%
Factory worker	41.4%	3.5%	28.2%
Commerce	4.0%	17.1%	8.6%
Construction worker	10.4%	0.9%	7.1%
Day laborer or domestic worker	9.5%	3.8%	7.5%
Agricultural worker	7.6%	3.2%	6.1%
Estimated daily wage (*n* = 987)			
Less than the minimum wage (lower than USD 9.3)	31.9%	74.2%	46.7%
Minimum wage	60.4%	18.8%	45.9%
More than the minimum wage	7.6%	7.0%	7.4%

*Acculturative stress*			
Duration of residence			
Less than 1 year	4.5%	6.1%	5.1%
1 to less than 5 years	44.6%	56.8%	48.8%
5 to less than 10 years	36.6%	24.9%	32.5%
10 years or more	14.3%	12.2%	13.6%
Have a Thai work permit (*n* = 987)	79.8%	6.9%	54.7%
Capacity to speak Thai (*n* = 987)			
None	44.7%	41.4%	43.6%
Weak	29.1%	37.1%	31.9%
Moderate	17.8%	14.2%	16.5%
Fluent	8.4%	7.2%	8.0%

*Social support on health care access *			
Receive support from family	73.4%	44.1%	63.1%
Receive support from friends	15.6%	5.8%	12.2%
Receive support from employers	7.0%	10.7%	8.3%
Receive support from health staff	10.7%	17.4%	13.1%

*MCH Access*			
Obtained or sought ANC for the last pregnancy	85.2%	73.2%	79.9%
Obtained or sought PPC after the last delivery	51.7%	79.9%	64.0%
Able to take their child to either health center or government hospital, if they become sick	97.0%	73.7%	88.5%

*Demographic factors of children*			
Child's age			
0–5 years	44.0%	52.5%	46.9%
6–10 years	34.4%	28.7%	32.4%
more than 10 years	21.6%	18.8%	20.6%
Child living arrangement (*n* = 987)			
Lives with both parents	88.9%	91.0%	89.7%
Lives with single mother	5.8%	4.3%	5.3%
Lives with single father	5.3%	4.6%	5.1%

**Table 2:** Multivariate logistic regression analysis of factors related to obtaining ANC during the last pregnancy, among GMS migrant workers whose youngest child is aged 0–4 years.

	Adjusted odds ratio	*p*
(1) Acculturative stress		
Country of origin		
Myanmar	0.92	0.88
Cambodia (reference)		
Duration of residence	1.01	0.73
Capacity to speak Thai		
Weak	0.95	0.88
Moderate	1.44	0.49
Fluent	7.93	0.02
None		
Have a Thai work permit	1.76	0.33

(2) Social support		
Social support on health access	12.17	0.00

(3) demographic factors		
Male	1.51	0.41
Age	0.96	0.10
Marital status		
Married, spouse present	0.83	0.87
Married, living separate	1.52	0.74
Divorced or widowed (reference)		

(4) Socioeconomic factors of respondents		
Occupation		
Deep-sea or coastal fisherman	0.21	0.18
Fisheries-related worker	0.31	0.29
Factory worker	0.40	0.41
Commerce	0.10	0.08
Construction worker	0.21	0.30
Day laborer or domestic worker	0.45	0.52
Agricultural worker		
Level of Education		
Some primary	3.31	0.02
Primary	2.39	0.09
Secondary or higher	3.84	0.03
No schooling		
Estimated daily wage		
Less than the minimum wage (<USD 9.3)	0.95	0.94
Minimum wage	0.88	0.86
More than the minimum wage		

Constant	1.254542	0.902157
−2 Log likelihood	257.35	
Nagelkerke *R* Square	0.42	
*N*	375	

**Table 3:** Multivariate logistic regression analysis of factors related to receiving PPC after the last delivery for GMS migrant workers whose youngest child is aged 0–4 years.

	Model 1		Model 2	
	Adjusted odds ratio	*p*	Adjusted odds ratio	*p*
(1) Acculturative stress				
Country of origin				
Myanmar	0.10	0.00	0.46	0.42
Cambodia (reference)				
Duration of residence	1.00	0.68	1.00	0.61
Capacity to speak Thai				
Weak	1.50	0.22	1.55	0.20
Moderate	1.63	0.23	1.71	0.19
Fluent	2.94	0.05	3.03	0.04
None				
Have a Thai work permit	0.67	0.39	0.61	0.28

(2) Social support				
Social support on health access	5.06	0.00	2.83	0.00
Country of origin *∗* Social support (Interaction)				
Cambodia at least 1 support			10.58	0.00
Myanmar at least 1 support			1.77	0.48
no support				

(3) Demographic factors				
Male	2.31	0.13	2.29	0.13
Age	0.99	0.72	0.99	0.69
Marital status				
Married, spouse present	5.17	0.10	4.21	0.15
Married, living separate	3.25	0.29	2.58	0.40
Divorced or widowed (reference)				

(4) Socioeconomic factors of respondents				
Occupation				
Deep-sea or coastal fisherman	0.22	0.03	0.27	0.07
Fisheries-related worker	0.30	0.04	0.32	0.06
Factory worker	0.43	0.14	0.46	0.16
Commerce	0.21	0.02	0.19	0.02
Construction worker	0.10	0.02	0.12	0.03
Day laborer or domestic worker	1.02	0.98	1.01	0.99
Agricultural worker				
Level of Education				
Some primary	1.37	0.50	1.24	0.66
Primary	1.68	0.27	1.63	0.33
Secondary or higher	2.48	0.09	2.52	0.10
No schooling				
Estimated daily wage				
Less than the minimum wage (<USD 9.3)	1.81	0.27	1.82	0.28
Minimum wage	3.05	0.09	3.19	0.09
More than the minimum wage				

Constant	0.20	0.26	0.07	0.07
−2 Log likelihood	361.68		348.881	
Nagelkerke *R* Square	0.39		.426	
*N*	375		375	

**Table 4:** Multivariate logistic regression analysis of factors related to receiving child health access among GMS migrant workers.

	Adjusted odds ratio	*p*
(1) Condition of acculturative stress of parents		
Country of origin		
Myanmar	3.043	0.051
Cambodia		
Duration of residence	1.004	0.296
Capacity to speak Thai (*n* = 994)		
Weak	.950	0.898
Moderate	.437	0.164
Fluent	1.483	0.606
None		
Have a Thai work permit	2.483	0.122

(2) Social Support		
Social support on health access	99.578	0.000

(3) Demographic factors		
Age of child		
Less than 1 year	4.878	0.181
1–5 years	.758	0.621
6–10 years	1.695	0.338
11–15 years	3.320	0.097
More than 15 years		
Child living arrangement		
Lives with both mother and father	5.454	0.013
Lives with only the mother	3.995	0.150
Lives with only the father		

(4) Socioeconomic factors of parents		
Occupation		
Deep-sea or coastal fisherman	.055	0.024
Fisheries-related worker	.165	0.135
Factory worker	.035	0.010
Commerce	.076	0.047
Construction worker	.342	0.560
Day laborer or domestic worker	.244	0.321
Agricultural worker		
Education recode-type 2 (4 levels)		
Some primary	.450	0.097
Primary	1.959	0.180
Secondary or higher	1.196	0.768
No schooling		
Estimated daily wage		
Less than the minimum wage (<USD 9.3)	2.182	0.232
Minimum wage	2.647	0.151
More than the minimum wage		

Constant	.166	0.250
−2 Log likelihood	263.1	
Nagelkerke *R* Square	0.67	
*N*	913	

## References

[1] Hunzai K., Gerlitz J. Y., Hoermann B. (2011). *Understanding Mountain Poverty in the Hindu Kush-Himalayas Regional report for Afghanistan, Bangladesh, Bhutan, China, India, Myanmar, Nepal, and Pakistan*.

[2] UNDP (2011). *Integrated Household Living Conditions Survey in Myanmar (2009-2010) - MDG Data*.

[3] Silber J., Wan G. (2016). *The Asian ‘Poverty Miracle’*.

[4] Sciortino R., Punpuing S. (2009). *International Migration in Thailand 2009*.

[5] Vapattanawong P., Chamratrithirong A., Punpuing S., Rhucharoenpornpanich O., Aphipornchaisakul K. (2016). Size and Distribution of Cross-Border Population from Myanmar, Cambodia, and Lao PDR in Thailand 2015: Estimation from Multiple Sources. *Thai Population Journal*.

[6] Chamratrithirong A., Punpuing S., Holumyong C., Chamchan C., Apipornchaisakul K., Kaikeaw S., Punpuing S., Holumyong C., Chunwan S. (2016). The Typology of Social Integration of Cross Border MW in Thailand. *Thailand in the Era of. Transnational Migration*.

[7] Walsh J., Ty M. (2011). Cambodian migrants in Thailand: Working conditions and issues. *Asian Social Science*.

[8] Kusakabe K., Pearson R. (2010). Transborder Migration, Social Reproduction and Economic Development: A Case Study of Burmese Women Workers in Thailand. *International Migration*.

[9] Guinto R. L. L. R., Curran U. Z., Suphanchaimat R., Pocock N. S. (2015). Universal health coverage in 'One ASEAN': Are migrants included?. *Global Health Action*.

[10] Isarabhakdi P. (2004). Meeting at the crossroads: Myanmar migrants and their use of Thai health care services. *Asian and Pacific Migration Journal*.

[11] Sabermahani A., Ghaderi H., Ashrafzadeh H. R. (2014). Patient Migration for Hospital Utilization: Case of Iran. *Health*.

[12] Baker S., Holumyong C., Thianlai K. (2010). *Research Gaps Concerning the Health of Migrants from Cambodia, Lao PDR and Myanmar in Thailand*.

[13] Asundep N. N., Carson A. P., Turpin C. A. (2013). Determinants of access to antenatal care and birth outcomes in Kumasi, Ghana. *Journal of Epidemiology and Global Health*.

[14] Sword W., Watt S., Krueger P. (2006). Postpartum health, service needs, and access to care experiences of immigrant and Canadian-born women. *JOGNN - Journal of Obstetric, Gynecologic, and Neonatal Nursing*.

[15] Nelson A. (2002). Unequal treatment: confronting racial and ethnic disparities in health care. *Journal of the National Medical Association*.

[16] Williams D. R., Rucker T. D. (2000). Understanding and addressing racial disparities in health care. *Health Care Financing Review*.

[17] Betancourt J. R., Green A. R., Carrillo J. E., Ananeh-Firempong O. (2003). Defining cultural competence: A practical framework for addressing racial/ethnic disparities in health and health care. *Public Health Reports*.

[18] LaVeist T. A. (1994). Beyond dummy variables and sample selection: What health services researchers ought to know about race as a variable. *Health Services Research*.

[19] Tielsch J. M., Sommer A., Witt K., Katz J., Royall R. M. (1990). Blindness and Visual Impairment in an American Urban Population: The Baltimore Eye Survey. *JAMA Ophtalmology*.

[20] Ahmad W. I. U., Kernohan E. E. M., Baker M. R. (1989). Influence of ethnicity and unemployment on the perceived health of a sample of general practice attenders. *Journal of Public Health (United Kingdom)*.

[21] Ford K., Chamrathrithirong A. (2007). Sexual partners and condom use of migrant workers in Thailand. *AIDS and Behavior*.

[22] Greenhalgh T., Helman C., Chowdhury A. M. (1998). Health beliefs and folk models of diabetes in British Bangladeshis: a qualitative study. *British Medical Journal*.

[23] Hong Y., Li X., Stanton B. (2006). Too costly to be ill: Health care access and health seeking behaviors among rural-to-urban migrants in China. *World health population*.

[24] Glanz K., Rimer B. K., Viswanath K. (2008). *Health Behavior and Health Education: Theory, Research, and Practice*.

[25] Lanoy E., Mary-Krause M., Tattevin P. (2007). Frequency, determinants and consequences of delayed access to care for HIV infection in France. *Antiviral Therapy*.

[26] Peng Y., Chang W., Zhou H., Hu H., Liang W. (2010). Factors associated with health-seeking behavior among migrant workers in Beijing, China. *BMC Health Services Research*.

[27] Besaggio D., Fuselli S., Srikummool M. (2007). Genetic variation in Northern Thailand Hill Tribes: Origins and relationships with social structure and linguistic differences. *BMC Evolutionary Biology*.

[28] Young G. (1961). *The Hill Tribes of Northern Thailand: (a Socio-ethnological Report)*.

[29] Win N. N. (2012). The taron: One of the hidden groups of hill ethnic groups in Myanmar. *Universities Research Journal*.

[30] Minden M. (1997). Midwives for refugees. *World Health*.

[31] Maschuw K., Vogt T., Samuel S. (2009). "Filling the gaps: NGO-approved Self-Help in tribal East Maraland, Myanmar. *Sight and Life Magazine*.

[32] Redfield R., Linton R., Herskovits M. J. (1936). Memorandum for the study of acculturation. *American Anthropologist*.

[33] Berry J. W. (1997). Immigration, acculturation, and adaptation. *Applied Psychology*.

[34] Bhugra D. (2004). Migration, distress and cultural identity. *British Medical Bulletin*.

[35] Frisbie W. P., Cho Y., Hummer R. A. (2001). Immigration and the health of Asian and pacific islander adults in the United States. *American Journal of Epidemiology*.

[36] Yi J. K. (1994). Acculturation, access to care and use preventive health services by Vietnamese women. *Asian American and Pacific Islander journal of health*.

[37] Sentell T., Shumway M., Snowden L. (2007). Access to mental health treatment by English language proficiency and race/ethnicity. *Journal of General Internal Medicine*.

[38] Jang M., Lee E., Woo K. (1998). Income, Language, and Citizenship Status: Factors Affecting the Health Care Access and Utilization of Chinese Americans. *Health & Social Work*.

[39] Berry J. W., Chun K., Organista P., Marin G. (2002). Conceptual approaches to acculturation. *Acculturation: Advances in Theory, Measurement, and Applied Research*.

[40] Lee J.-S., Koeske G. F., Sales E. (2004). Social support buffering of acculturative stress: A study of mental health symptoms among Korean international students. *International Journal of Intercultural Relations*.

[41] Jitthai N. *Healthy Migrant, Healthy Thailand: A Migrant Health Program Model*.

[42] Swaddiwudhipong W., Ngamsaithong C., Peanumlom P., Hannarong S. (2008). An outbreak of cholera among migrants living in a Thai-Myanmar border area. *Journal of the Medical Association of Thailand*.

[43] Srithamrongsawat S., Wisessang R., Ratjaroenkhajorn S. (2010). *Financing Health Care for Migrants: A Case Study from Thailand*.

[44] Zimet G. D., Dahlem N. W., Yimet S. G., Farlez G. K. (1988). The multidimensional scale of perceived social support. *Journal of Personality Assessment*.

[45] Zimet G. D., Powell S. S., Farley G. K., Werkman S., Berkoff K. A. (1990). Psychometric characteristics of the multidimensional scale of perceived social support. *Journal of Personality Assessment*.

[46] Tonsing K., Zimet G. D., Tse S. (2012). Assessing social support among South Asians: The multidimensional scale of perceived social support. *Asian Journal of Psychiatry*.

[47] Aroian K., Templin T. N., Ramaswamy V. (2010). Adaptation and Psychometric Evaluation of the Multidimensional Scale of Perceived Social Support for Arab Immigrant Women. *Health Care for Women International*.

[48] Chamchan C., Apipornchaisakul K. (2012). *A Situation Analysis on Health System Strengthening for Migrants in Thailand*.

[49] Harris M., Jones D., Brookes S., Grant J. (1986). Relations between the non-verbal context of maternal speech and rate of language development. *British Journal of Developmental Psychology*.

[50] Chamratrithirong A., Boonchalaksi W. (2008). *Prevention of HIV/AIDS among Migrant Workers in Thailand Project (PHAMIT): The Impact Survey*.

